# Evaluation of Bi-Layer Silk Fibroin Grafts for Tubular Ureteroplasty in a Porcine Defect Model

**DOI:** 10.3389/fbioe.2021.723559

**Published:** 2021-09-17

**Authors:** Gokhan Gundogdu, Zhamshid Okhunov, Vivian Cristofaro, Stephanie Starek, Faith Veneri, Hazem Orabi, Pengbo Jiang, Maryrose P. Sullivan, Joshua R. Mauney

**Affiliations:** ^1^ Department of Urology, University of California, Irvine, Irvine, CA, United States; ^2^ Division of Urology, Veterans Affairs Boston Healthcare System, Boston, MA, United States; ^3^ Department of Surgery, Brigham and Women’s Hospital, Harvard Medical School, Boston, MA, United States; ^4^ Department of Biomedical Engineering, University of California, Irvine, Irvine, CA, United States

**Keywords:** biomaterials, silk fibroin, tissue engineering, ureter, scaffold

## Abstract

Ureteral reconstruction with autologous tissue grafts is often limited by tissue availability and donor site morbidity. This study investigates the performance of acellular, bi-layer silk fibroin (BLSF) scaffolds in a porcine model of ureteroplasty. Tubular ureteroplasty with BLSF grafts in combination with transient stenting for 8 weeks was performed in adult female, Yucatan, mini-swine (*N* = 5). Animals were maintained for 12 weeks post-op with imaging of neoconduits using ultrasonography and retrograde ureteropyelography carried out at 2 and 4 weeks intervals. End-point analyses of ureteral neotissues and unoperated controls included histological, immunohistochemical (IHC), histomorphometric evaluations as well as *ex vivo* functional assessments of contraction/relaxation. All animals survived until scheduled euthanasia and displayed mild hydronephrosis (Grades 1-2) in reconstructed collecting systems during the 8 weeks stenting period with one animal presenting with a persistent subcutaneous fistula at 2 weeks post-op. By 12 weeks of scaffold implantation, unstented neoconduits led to severe hydronephrosis (Grade 4) and stricture formation in the interior of graft sites in 80% of swine. Bulk scaffold extrusion into the distal ureter was also apparent in 60% of swine contributing to ureteral obstruction. However, histological and IHC analyses revealed the formation of innervated, vascularized neotissues with *a*-smooth muscle actin+ and SM22α+ smooth muscle bundles as well as uroplakin 3A+ and pan-cytokeratin + urothelium. *Ex vivo* contractility and relaxation responses of neotissues were similar to unoperated control segments. BLSF biomaterials represent emerging platforms for tubular ureteroplasty, however further optimization is needed to improve *in vivo* degradation kinetics and mitigate stricture formation.

## Introduction

Ureteral injuries caused by intraoperative endourological maneuvers, radiation exposure, or gynecological procedures can lead to life-threatening complications such as sepsis, urinary tract obstruction, and loss of renal function (Packiam et al., 2016; Blackwell et al., 2018). In addition, penetrating traumas from gunshot and stab wounds can also result in ureteral perforation with a mortality rate of 6% (Siram et al., 2010). Surgical management of short ureteral defects (<2 cm in length) is often performed with primary anastomosis following resection of damaged segments (Burks and Santucci, 2014), however for long ureteral pathologies, autologous tissue grafting is necessary to restore organ continuity (Mauck et al., 2011; Kocot et al., 2017). Traditional surgical approaches for ureteral repair deploying patient-derived donor tissues include uretero-ureterostomy, uretero-neocystostomy, Boari flap, transuretero-ureterostomy, renal autotransplantation, and ileal interposition with the choice of method being dependent on the size and location of injury (Simaioforidis et al., 2013). However, the success of these strategies can be compromised by donor site morbidity, reflux, stricture formation, hydronephrosis, and persistent anastomotic leakage (Engel et al., 2015; Xiong et al., 2020; Heijkoop and Kahokehr, 2021). These complications often necessitate secondary salvage procedures to maintain upper urinary tract function, but in the worst case scenario, nephrectomy may be required (Onal et al., 2018; Vasudevan et al., 2019). These reports highlight the need for novel strategies for ureteral reconstruction which can minimize unwanted side-effects and promote functional tissue regeneration.

Biodegradable 3-D scaffolds composed of decellularized tissues, collagen or synthetic polyesters have been previously investigated as potential substitutes to autologous tissue grafts for ureteral repair (Kloskowski et al., 2013; Simaioforidis et al., 2013; de Jonge et al., 2015). These biomaterials have been utilized alone or seeded with primary urothelial, smooth muscle, and/or mesenchymal stem cell populations for reconstruction of patch or tubular ureteral defects in preclinical animal models (El-Hakim et al., 2005; Liao et al., 2013; Meng et al., 2015; Xu et al., 2020). Despite the ability of these tissue engineered constructs to support constructive remodeling following onlay ureteroplasty (Liatsikos et al., 2001; Smith et al., 2002), suboptimal outcomes including stricture formation, hydronephrosis, graft contracture, and fibrosis have been reported following implantation of tubular scaffold configurations (Duchene et al., 2004; Janke et al., 2019). Currently, there is no FDA-approved tissue engineered device for ureteral tissue replacement. Advancements in ureteral tissue engineering are dependent on new scaffold designs which can preserve upper urinary tract function while promoting host regenerative responses.

Acellular, bi-layer silk fibroin (BLSF) grafts represent emerging options for urinary tract reconstruction due to their low immunogenicity, robust mechanical strength and elasticity, tunable biodegradability, and diverse processing plasticity (Sack et al., 2016). These protein-based, biomaterials derived from *Bombyx mori* silkworm cocoons can be fashioned into sheets or tubes and their bi-layer architecture prevents urinary extravasation at implant sites via a fluid-tight film layer, while a porous foam compartment allows for ingrowth of surrounding host tissues (Algarrahi et al., 2018a; Affas et al., 2019). In comparison to silk fibroin foams alone, the addition of the annealed film layer minimizes graft perforation and enhances suture retention strength during urinary tract reconstruction by increasing the ultimate tensile strength and elastic modulus of the bulk matrix (Tu et al., 2013). Previous reports from our laboratory have detailed the ability of BLSF grafts to promote improved functional performance, enhanced tissue regeneration, and minimal inflammatory reactions in respect to conventional SIS matrices across various preclinical models of bladder and urethral reconstruction encompassing both traumatic injury and pathologic settings (Chung et al., 2014a; Chung et al., 2014b). In addition, constructive remodeling of BLSF scaffolds has been shown to promote formation of innervated, vascularized urologic neotissues with contractile properties similar to native counterparts (Seth et al., 2013; Tu et al., 2013; Affas et al., 2019). In the present study, we evaluated the efficacy of tubular BLSF grafts to support functional tissue regeneration of ureteral defects in a porcine model of ureteroplasty.

## Materials and Methods

### Biomaterials

Aqueous silk fibroin solutions derived from *B. mori* silkworm cocoons were used to fabricate BLSF biomaterial sheets following previously described protocols (Seth et al., 2013). Briefly, an 8% weight/volume silk fibroin solution was dried in a casting vessel under a laminar flow hood for 48 h to produce a silk fibroin film. A 6% weight/volume silk fibroin solution was then combined with sieved granular NaCl (500–600 μM, average crystal diameter) in a ratio of 2 g NaCl per ml of silk fibroin solution and layered on top of the silk fibroin film. The resultant solution was allowed to solidify and fuse to the silk fibroin film for 48 h at 37 °C to create the BLSF matrix. NaCl was removed thereafter by washing the scaffold for 72 h in distilled water with regular volume changes. Structural and mechanical properties of BLSF matrices have been published in past reports (Algarrahi et al., 2018b). In particular, the BLSF biomaterial was composed of a foam compartment consisting of interconnected pores (∼400 μm diameter) buttressed by a homogeneous, non-porous film layer (∼200 μm thick). Uniaxial tensile properties of the BLSF graft were previously determined as ultimate tensile strength: 0.3 ± 0.1 MPa; elastic modulus: 3.6 ± 1.3 MPa; and elongation to failure: 24.7 ± 8.9% (Algarrahi et al., 2018b). BLSF matrix sheets were fashioned into tubular implants (2 cm in length, 1 cm in outer diameter, 0.6 cm in inner diameter) using 4-0 propylene sutures. Biomaterials were steam sterilized in an autoclave at 120°F for 20 min prior to implantation.

### Surgical Procedures

All animal husbandry, surgical manipulations, and imaging evaluations followed guidelines prescribed by the National Institutes of Health’s Guidelines for the Care and Use of Laboratory Animals and were reviewed and approved by the University of California, Irvine Animal Care and Use Committee and performed under protocol AUP-19-166. This study was also conducted in compliance with ARRIVE guidelines (https://arriveguidelines.org).

**FIGURE 1:**
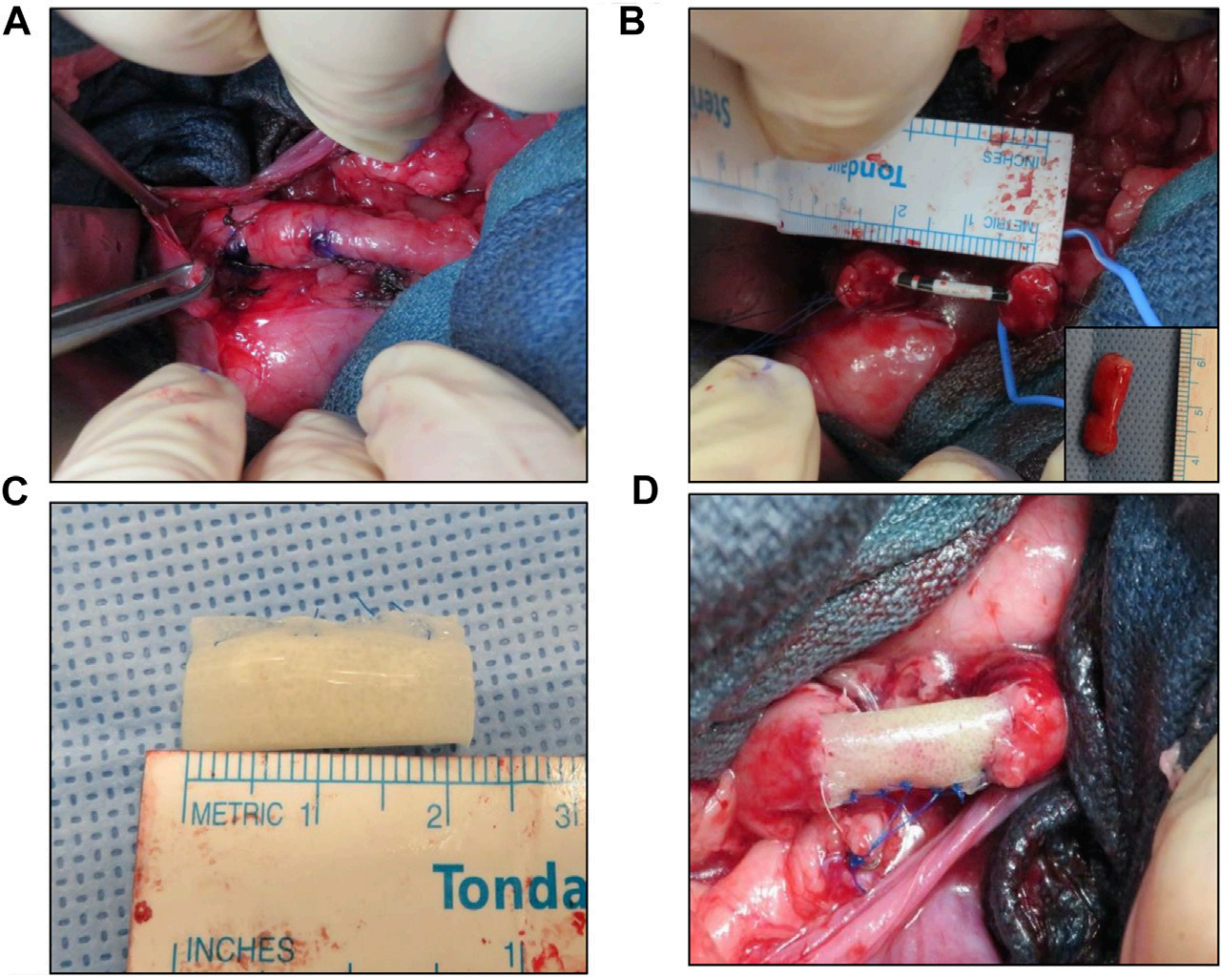
**Porcine tubular ureteroplasty model. (A)** Exposure of proximal ureter and marking of target reconstructive area. **(B)** Creation of tubular ureteral defect and stent placement. Inset denotes excised tissue. **(C)** Tubular BLSF graft prior to implantation. **(D)** BLSF graft anastomosed into ureteral defect. BLSF, bi-layer silk fibroin scaffold.

Tubular ureteroplasty was performed in five adult female, Yucatan mini-swine (∼24 weeks of age, 30–40 kg, Premier BioSource, Ramona, CA) using either a retroperitoneal flank (*N* = 4) or transperitoneal (*N* = 1) approach (Figure 1). Male swine were excluded from the study since catheter placement into the urinary tract cannot be performed reliably due to excessive tortuosity of the male urethra. Prior to operative procedures, swine were fasted 12 h with free access to water. General anesthesia was initiated by intramuscular injection of 0.4 mg/kg atropine, 2.2 mg/kg Anased (Lloyd Inc.; IA, United States), and 4.4 mg/kg Telazol (Zoetis Inc.; Parsippany, NJ, United States), and maintained with 1–4% isoflurane inhalation through an endotracheal tube. A retrograde ureteropyelogram (RUPG) was first performed via catheterization to confirm the native anatomy of the distal right ureter. The catheter was left inside the ureter to aid in surgical manipulations.

Swine were maintained in the supine position for the transperitoneal approach or were repositioned to the left lateral decubitus for the retroperitoneal flank procedure. Following skin incision, the abdominal wall layers were separated allowing entry into the retroperitoneal space. A 2 cm segment of the right upper ureter ∼5 cm below the pelvi-ureteric junction was resected without compromising the ureteral vasculature. The ureteral catheter was then replaced by a six French, double pigtail ureteral stent (Inlay Optima; BARD Inc., Covington, GA, United States) using a hybrid guidewire (Boston Scientific, Boston, MA, United States). A tubularized BLSF implant (2 cm in length) was positioned in line within the ureteral defect and over the ureteral stent. The graft was then anastomosed to the surrounding host tissues using interrupted 5-0 monofilament polyglactin sutures. Proximal and distal boundaries of the implant with host ureter were marked with non-absorbable 4-0 polypropylene sutures (Ethicon, Somerville, NJ, United States). In addition, proximal/distal radiopaque marking rings were placed adjacent to the implant site to identify the graft area during fluoroscopic evaluations. A Penrose drain was placed into the retroperitoneum in Pigs 2–5 to drain any excess fluid over the course of 7 days and removed thereafter. The abdominal incision was closed in separate layers and a Foley catheter was introduced into the bladder. The Foley catheter was fixed to the skin with non-absorbable sutures and removed after 5 days. For postoperative pain management, all animals received subcutaneous infiltration of 1–3 mg/kg Bupivacaine HCl 0.25% (Hospira Inc.; Lake Forest, IL, United States) after abdominal wound closure, an intramuscular injection of 2 mg/kg Banamine (Merck Animal Health; Kenilworth, NJ, United States) and subcutaneous injection of 0.15 mg/kg Buprenorphine SR (ZooPharm; Laramie, WY, United States) for 72 h. Animals were recovered in warm blankets until they became sternal. Enrofloxacin (Baytril, Bayer Healthcare LLC, KA, United States) was intramuscularly administered (7.5 mg/kg) prior to each invasive procedure and was continued orally for 4 days post-op.

Following 8 weeks post-op, the ureteral stent was removed from the right ureter (except for Pig 2) by using a flexible cystoscope (Model #: 11272C; Karl Storz, Tuttlingen, Germany) and endoscopic grasper (Model#:11,275 FE, Karl Storz, Tuttlingen, Germany) to evaluate neoureter and kidney function without urinary diversion. Longitudinal imaging evaluations were performed prior to surgical manipulations and at 1, 2, 4, 8, and 12 weeks post-op to monitor ureter continuity, kidney morphology, and indwelling catheter position. These analyses included ultrasonography (USG), RUPG and video-endoscopy (cystoscopy, ureterorenoscopy) as described in the following sections.

All animals were maintained for a total of 12 weeks and then sacrificed with an intravenous 0.2 ml/kg pentobarbital sodium and phenytoin sodium euthanasia solution (Euthasol; Virbac AH, Westlake, TX, United States). Following necropsy, reconstructed ureteral segments were excised from host tissues and divided axially into four circumferential rings (∼4-5 mm in length) representing two peripheral (adjacent to anastomotic border) and two central zones of neotissues. Tubular specimens from both central and peripheral regions were evaluated by histological, immunohistochemical (IHC), and histomorphometric analyses as well as for *ex vivo* assessment of functional responses to contractile/relaxing agents as detailed below. Tubular ureteral segments excised either 5 cm above or below the scaffold implantation site were evaluated in parallel as internal proximal controls (IPC) or internal distal controls (IDC), respectively. In addition, native ureteral tissues that were either excised to create the initial defect for graft implantation or harvested from the unoperated ureters were assessed similarly as nonsurgical controls (NSC).

### Urinary Tract Ultrasonography

USG imaging was performed under general anesthesia by using Preirus Ultrasound system (Hitachi Aloka, Mitaka-shi, Tokyo, Japan) outfitted with a standard 10 Hz transducer probe to examine the right kidney parenchyma, hydronephrosis, perirenal collection, catheter position, and left kidney for reference. The severity of hydronephrosis was evaluated using the Onen hydronephrosis grading system on a scale from 0 to 4 as previously described (Onen, 2007).

### Cystoscopy, Retrograde Ureteropyelogram, and Ureterorenoscopy

A Cysto-Urethro-Fiberoscope (Model no: 11,272 C; Karl Storz, Tuttlingen, Germany) was used for cystoscopy, catheter manipulations, and contrast studies. Under general anesthesia, swine were placed in supine position, scrubbed, and sterilely draped. The cystoscope was gently introduced into the bladder with saline infusion provided through the working channel. For contrast imaging, the distal right ureter was catheterized with a hybrid working wire (Sensor™; Boston Scientific, MA, United States) introduced through the working channel of the scope. A retrograde uretero-pyelogram [1:1 diluted iohexol contrast agent (Omniopaque 300; GE Healthcare, Milwaukee, WI, United States)] was performed with a five French open-ended catheter (Cook Urological Inc.; Bloomington, IN, United States) to demonstrate the renal collecting system with anterior/posterior and lateral images acquired with C-arm fluoroscopy (BV Pulsera; Philips, Eindhoven, Netherlands). Neotissues were identified with radiopaque markers positioned at the time of grafting. URS was performed at 12 weeks post-op prior to sacrifice to examine the extent of mucosal healing by using a flexible ureterorenoscope (Flex-X2S; Karl Storz, Tuttlingen, Germany). Video and still images were captured by a video processor system (Image 1 HUB; Karl Storz, Tuttlingen, Germany).

### *Ex vivo* Tissue Contractility and Relaxation Responses

Ureteral tissue specimens (*N* = 4 per group) were excised from the peripheral and central regions of BLSF graft sites and IPC segments at 12 weeks post-op and *ex vivo* contractility/relaxation responses were evaluated and compared to NSC as follows. After tissues were dissected into strips (6–8 mm in length and 2–3 mm in width) and denuded of mucosa, specimens were transferred to 5 ml organ chambers containing Kreb’s solution maintained at 37°C by a thermoregulated water circuit and continuously bubbled with a mixture of 95% O_2_ and 5% CO_2_ (pH at 7.4). Each strip was tied at both ends by silk threads and connected to a force-displacement transducer to measure isometric tension from the circular muscle layer. Tissue samples were stretched to a force of 2 g and equilibrated for at least 120 min. After the equilibration period, amplitude and frequency of spontaneous rhythmic contractions were recorded for a period of 30 min (baseline conditions). Increasing doses of phenylephrine (PE, 0.1–300 µM) were administered to the chambers and the resulting phasic contractions (amplitude and frequency) were measured. Following the last concentration of PE, a single dose of isoproterenol (ISO, 10 μM) was added to provoke relaxation. The amplitude of contractile responses to the muscarinic receptor agonist, carbachol (CCh, 10 µM), was also evaluated. Receptor independent, smooth muscle contractions were elicited by exposure to a 120 mM KCl solution. Electrical field stimulation (30V, 1 ms duration, 1–20 Hz), provided by parallel platinum electrodes embedded in the organ bath, was delivered at 2 min intervals to generate frequency-response curves. At the end of the experiment, the length, width and weight of each strip were recorded to normalize the force measurements by tissue cross-sectional area.

### Histological, Immunohistochemical, and Histomorphometric Analyses

Tubular ureteral specimens from central and peripheral regions of neotissues (*N* = 5), IPC (*N* = 5), IDC (*N* = 5), and NSC were excised from swine following sacrifice, fixed in 10% neutral-buffered formalin, subjected to alcohol dehydration, and embedded in paraffin. Specimens were sectioned (5 μm), stained with Masson’s Trichrome (MTS), digitally imaged across the entire section, and collagen content was determined with a color segmentation program in ImageJ using previously described protocols to quantify blue-stained color elements indicative of collagen deposition (Affas et al., 2019). Collagen content was calculated as the percentage of the blue stained area (collagen) per total field area evaluated and normalized to NSC levels.

Immunohistochemical (IHC) assessments were performed on parallel tissue sections after antigen retrieval in 10 mM sodium citrate buffer (pH 6.0) and incubation in blocking buffer consisting of phosphate-buffered saline with 5% fetal bovine serum, 1% bovine serum albumin, and 0.3% Triton X-100 for 1 h at room temperature. Specimens were probed with the following primary antibodies overnight at 4°C: anti-pan-cytokeratin (CK) (1:150 dilution; Dako, Carpinteria, CA), anti-α-smooth muscle actin (SMA) (1:200 dilution; Sigma-Aldrich, St. Louis, MO), anti-SM22α (1:200 dilution, Abcam, Cambridge, MA), anti-S100 (1:200 dilution, Abcam), anti-uroplakin (UP) 3A (1:10 dilution, Fitzgerald, North Acton, MA), and anti-CD31 (1:100 dilution; Abcam). Specimens were then stained with species-matched Alexa Fluor 594-conjugated secondary antibodies (Thermo Fisher Scientific, Waltham, MA) and nuclei were counterstained with 4′, 6-diamidino-2-phenyllindole (DAPI). Specimen visualization was performed with a Zeiss Axio Imager M2 model (Carl Zeiss MicroImaging, Thornwood, NY) and representative fields were acquired with Zen software (version 3.1). Negative controls consisting of parallel specimens stained with secondary antibodies in the absence of primary antibodies were performed similarly and produced no detectable signal above background.

Histomorphometric evaluations (*N* = 5 swine per cohort) were carried out on four independent microscopic fields (10X magnification) equally spaced along the circumference of each reconstructed and control tubular specimen using previously published protocols (Affas et al., 2019). Specifically, area measurements and image thresholding were performed on microscopic fields with ImageJ software (version 1.47) to determine the percentage of tissue area stained for *a*-SMA, SM22α and pan-CK per total field area evaluated. The number of CD31^+^ vessels were calculated across four independent microscopic fields (10X) per sample using similar methods and normalized to total field area to calculate vascular density.

### Statistical Analysis

Statistical evaluations of quantitative data were carried out using the Kruskal–Wallis test in combination with post hoc Dunn’s test for pairwise comparisons, considering a value of *p* < 0.05 as significant. Quantitative data were displayed as mean ± standard deviation (SD) or ± standard error (SE) when specified.

## Results

**FIGURE 2:**
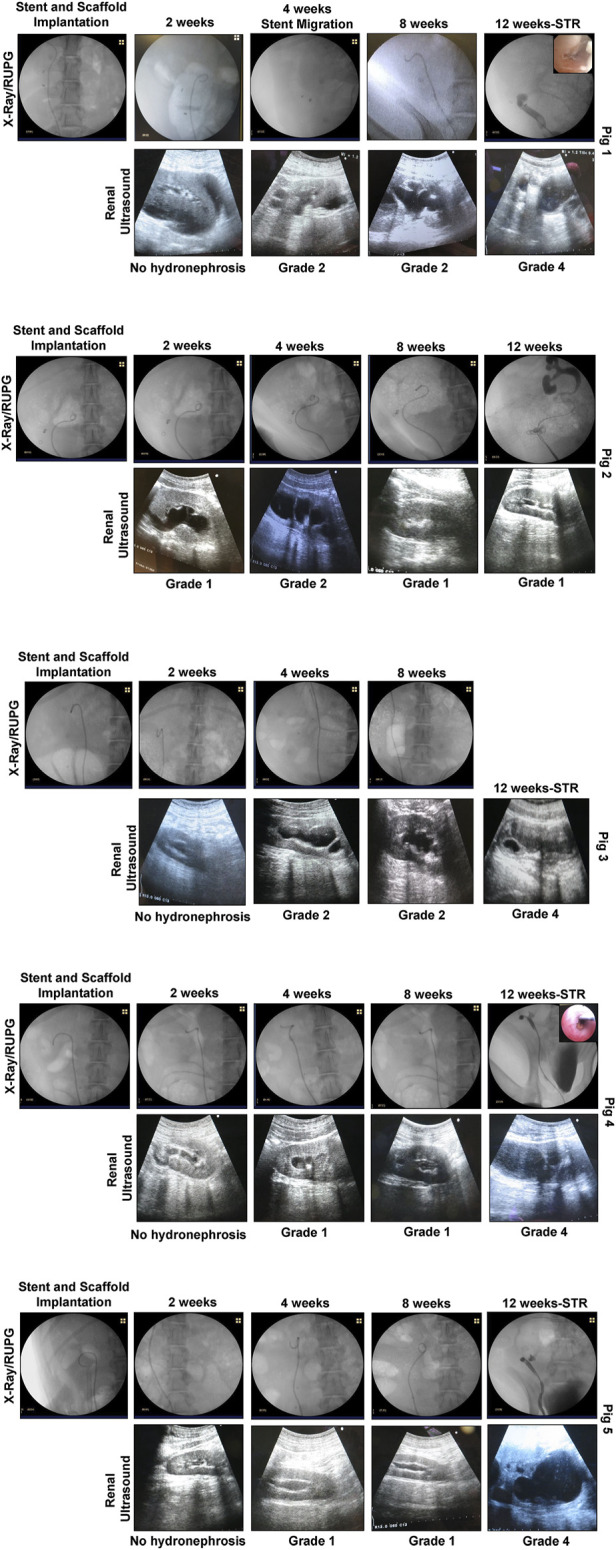
***In situ* imaging evaluations of reconstructed ureteral conduits.** Representative data for Pigs 1–5. Ureters were implanted with stented, tubular BLSF matrices. Stents were removed at 8 weeks post-op and swine were harvested at 3 months. In the majority of swine, severe Grade 4 hydronephrosis and neotissue stricture formation were detected in operated ureters at sacrifice. X-Ray (2, 4, 8 weeks) and contrast RUPG (12 weeks) (top rows); sonograms (bottom rows with hydronephrosis scores); and URS (insets showing regenerated mucosa) assessments were performed at various experimental timepoints. STR, stricture and RUPG, retrograde ureteropyelogram.

Tubular ureteroplasty with BLSF grafts was performed in five swine in combination with transient ureteral stenting of the implantation site to reinforce initial neoconduit integrity (Table 1). Anastomotic leak testing following ureteroplasty demonstrated a fluid-tight anastomosis during all five procedures. Fluoroscopic evaluations (Figure 2) confirmed the proximal coil of the double pigtail ureteral stent was properly positioned in the renal pelvis while the distal end was maintained in the bladder in each case. No signs of hydronephrosis were detected in any swine at time of ureteroplasty confirming normal anatomy of the collecting system. There were no intraoperative or immediate postoperative complications encountered in swine repaired with either the retroperitoneal flank or transperitoneal approach and all animals were successfully recovered from anesthesia.

**TABLE 1:** **Surgical Outcomes of Tubular Ureteroplasty.** Representative data from Pigs 1–5.

Animal	Ureteroplasty approach and stent strategy	Ultrasonography (hydronephrosis grade)				Complications and management	Terminal outcomes
		2 weeks	4 weeks	8 weeks	12 weeks		
Pig 1	Transperitoneal + double pigtail stent for 8 weeks	No	2	2	4	Stent migration into the bladder at 4 weeks post-op. New stent reinserted until 8 weeks timepoint.	Stricture found in central neotissue. Bulk scaffold extruded into distal ureteral lumen
Pig 2	Retroperitoneal flank + double pigtail stent for 12 weeks	1	2	1	1	Subcutaneous fistula formation at 2 weeks	Fistula formation in central neotissue
Pig 3	Retroperitoneal flank + double pigtail stent for 8 weeks	No	2	2	4	None	Stricture found in central neotissue. Bulk scaffold extruded into distal ureteral lumen
Pig 4	Retroperitoneal flank + double pigtail stent for 8 weeks	No	1	1	4	None	Stricture found in central neotissue.
Pig 5	Retroperitoneal flank + double pigtail stent for 8 weeks	No	1	1	4	None	Stricture found in central neotissue. Bulk scaffold extruded into implant site.

At 1 week post-op, all swine had their Penrose drains and Foley catheters removed. There were no signs of surgical site infection or hydronephrosis at this timepoint and all animals were capable of normal micturition for the duration of the study following removal of the Foley catheter. Following 2 weeks of scaffold implantation, radiographic and sonographic assessments (Figure 2) on all five animals confirmed stent position in the urinary tract with no substantial evidence of hydronephrosis or renal abnormalities observed in any case. However, urine discharge was noted in Pig 2 from the subcutaneous closure area which was left to heal spontaneously. Imaging evaluations at 4 weeks post-ureteroplasty confirmed stent position in all swine except Pig 1 wherein the stent was dislodged into the urinary bladder. Cystoscopy was performed to remove the migrated stent from the bladder and a new stent was inserted as described above under radiographic guidance. Mild hydronephrosis (Grades 1-2) was observed in the reconstructed collecting systems of all animals at this timepoint with dilation of the renal pelvis, but no evidence of renal parenchymal damage. Urine drainage from the cutaneous wound closure area was still observed in Pig 2 in the absence of erythema, tenderness, or other signs of infection.

At 8 weeks post-op, ureteral stents were removed from the urinary tract in all swine except Pig 2 which displayed a persistent urine discharge from the primary surgical incision area. RUPG and fistulographic analyses confirmed the presence of an uretero-cutaneous fistula in close proximity to the reconstructed site in Pig 2. Imaging evaluations (Figure 2) of all swine at this stage of repair revealed an unobstructed lumen at the graft site with mild hydronephrosis (Grades 1-2) of the reconstructed upper urinary tract observed in all cases similar to the findings at the 4 weeks timepoint. The ability of ureteral neotissues to support upper urinary tract function without stenting was assessed over the course of a 4 week period. At 12 weeks post-op, radiographic and sonographic assessments (Figure 2) demonstrated obstruction of the original graft area with severe hydronephrosis (Grade 4) in the reconstructed collecting systems of all unstented swine (Pigs 1, 3–5). In contrast, continuously stented ureteral tissue in Pig 2 resulted in mild hydronephrosis (Grade 1) and free passage of contrast material through the neotissue. URS surveillance demonstrated mucosal regeneration at the distal graft region in the majority of animals, however stricture formation was apparent in the center of all neotissues except Pig 2.

**FIGURE 3:**
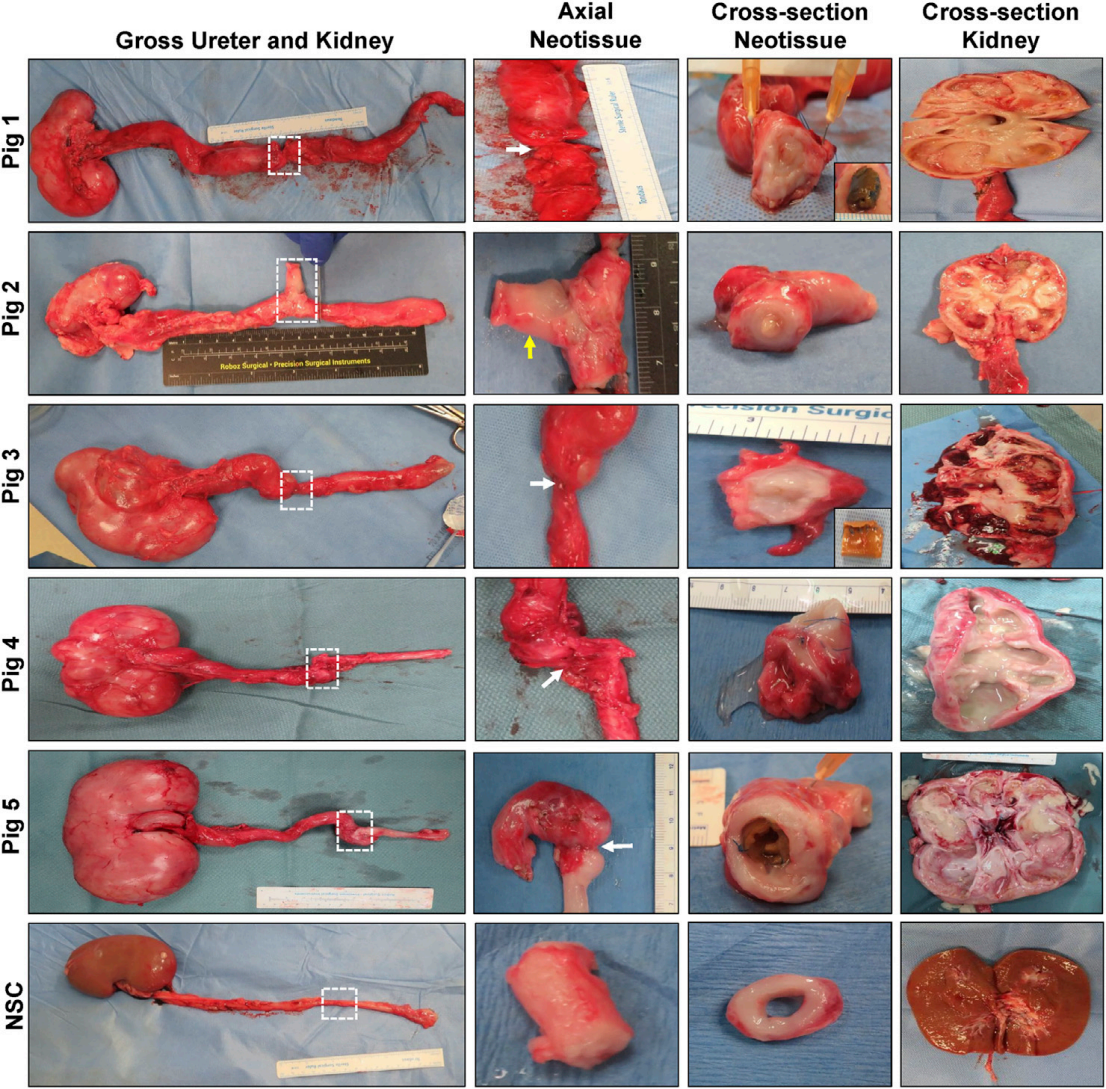
**Necropsy evaluations of ureteral neotissues and reconstructed collecting systems.** First column: Photomicrographs of gross collecting systems and reconstructed areas (boxed) in Pigs 1–5 after 12 weeks of scaffold implantation as well as parallel nonsurgical controls (NSC). Second and third columns display axial and cross-sectional views of neoconduits from original graft sites, respectively. White arrows denote central stricture formation in neotissues and the yellow arrow marks fistula formation. Insets display bulk scaffold remnants isolated from the distal ureter. Cross-sectional view of kidneys from operated collecting systems or NSC are shown in the fourth column.

Following scheduled euthanasia, necropsy assessments revealed host tissue ingrowth throughout the original implantation site in all swine with minimal abdominal adhesions observed on the exterior of neotissues (Figure 3). Regenerated ureteral tissues exhibited minimal axial contraction between the proximal/distal marking sutures, however the presence of strictures at the central graft site in Pigs 1, 3–5 confirmed URS findings. In addition, the formation of an uretero-cutaneous fistula was confirmed in the central neotissue region of Pig 2. Tubular BLSF grafts remained largely intact in the reconstructed collecting system and were either found extruded in the lumen of the graft site (Pig 5) or displaced into the unoperated segment of the distal ureter (Pigs 1, 3). Gross inspection of the reconstructed collecting systems confirmed imaging findings and demonstrated severe hydronephrosis in Pigs 1, 3–5 with substantial dilation of renal calyces and pelvis consistent with urine outflow obstruction. These results were in contrast with the unoperated collecting system which displayed normal anatomy.

**FIGURE 4:**
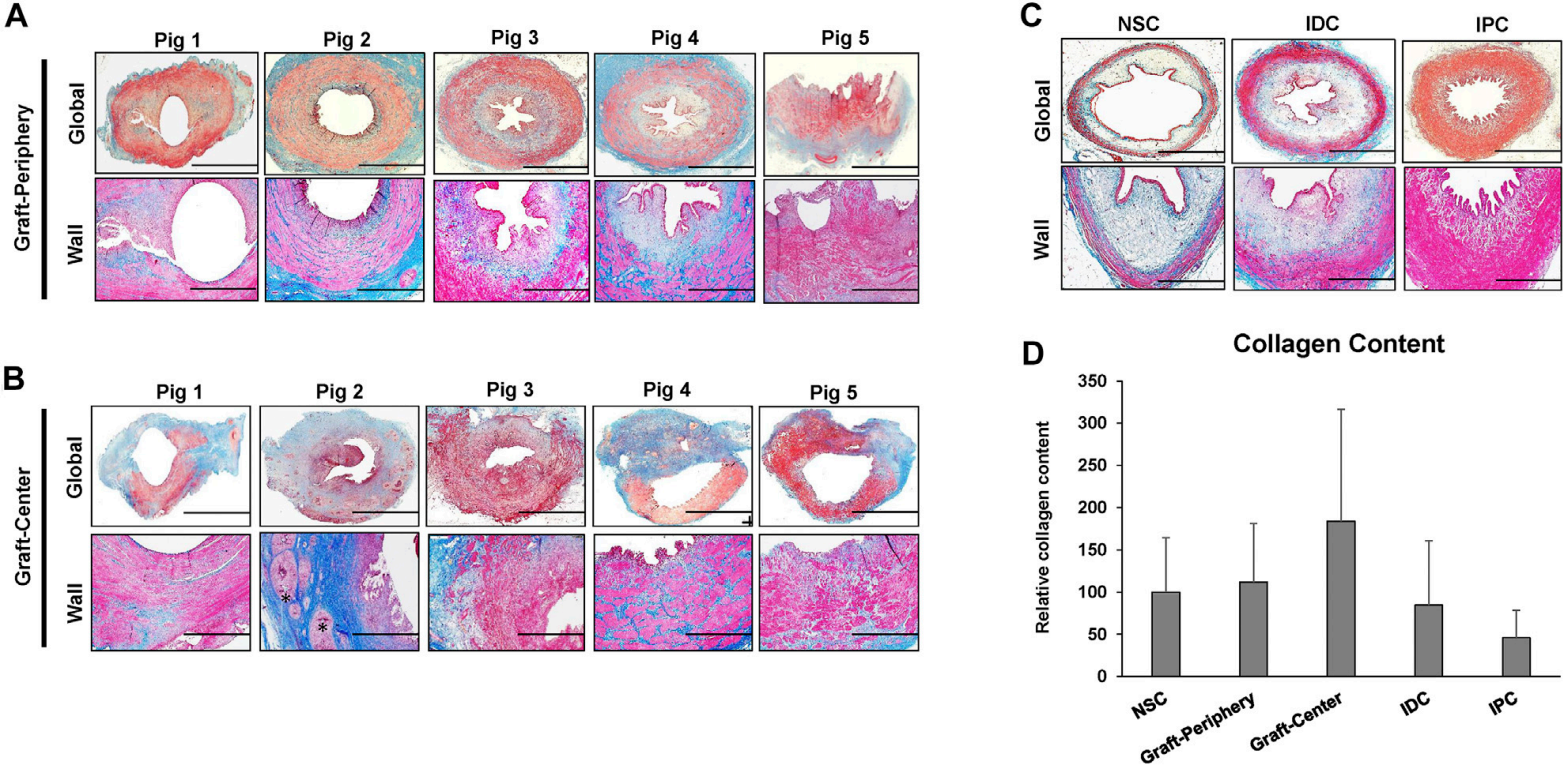
**Histological assessments of ureteral tissue architecture in controls and neotissues.** Photomicrographs of peripheral **(A)** and central **(B)** areas of reconstructed tissues from all swine as well as control groups consisting of NSC, IPC, and IDC segments procured from Pig 5 **(C)** stained with Masson’s trichrome. Gross cross-sections of ureteral tissues are presented in top rows with magnified views of the ureteral wall displayed in the second row of panels. Scale bars for 1^st^ and 2^nd^ rows in each panel are 1 cm and 400 μm, respectively. A semi-circumferential ureteral section is displayed for the graft-periphery of Pig 5. Asterisk denotes residual scaffold fragments in A-B. **(D)** Quantitation of collagen content in MTS-stained specimens displayed in **(A–C)**. For all panels, *N* = 5 animals per group were assessed per data point. For panel D, data were analyzed with Kruskal–Wallis test. *p* = 0.326. Values are displayed as means ± SD. NSC, nonsurgical controls; IPC, internal proximal controls; IDC, internal distal controls.

Histological (MTS) evaluations (Figure 4) of study groups revealed that the cross-sectional organization of both peripheral and central neotissues demonstrated architecture similar to NSC with a multi-layer urothelium, a vascularized lamina propria, and an outer layer of smooth muscle bundles. However, in comparison to NSC, smooth muscle hypertrophy and hyperplasia were apparent in all reconstructed neotissues as well as in IPC and IDC segments consistent with compensatory mechanisms of ureteral obstruction. In addition, central neotissue regions displayed a discontinuous layer of smooth muscle bundles and substantially elevated levels of collagen content in respect to other experimental groups suggesting ongoing tissue remodeling. Moreover, scattered mononuclear inflammatory cells were also observed surrounding minute scaffold fragments in the center of neoconduits consistent with an active stage of wound healing.

**FIGURE 5:**
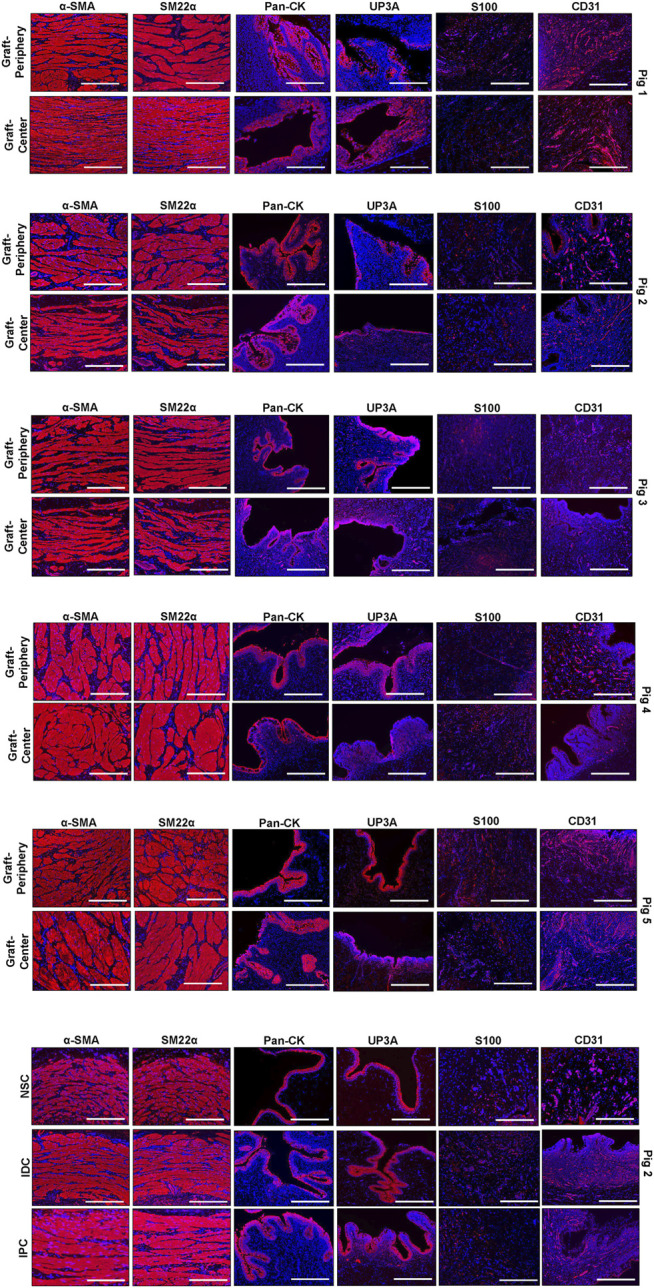
**Immunohistochemical evaluations of neconduit maturation and control tissues.** Representative photomicrographs of selective protein expression in peripheral and central regions of neotissues at 12 weeks post-op as well as in control groups: NSC, IPC, and IDC. Markers of interest include smooth muscle contractile proteins (α-SMA, SM22α), epithelial proteins (pan-CK, UP3A), as well as vascular (CD31) and innervation (S100) proteins. For all panels, respective marker expression is labeled in red (Alexa Fluor 594 labeling) with DAPI nuclear counterstain displayed in blue. Scale bars = 400 µm in each panel. Representative results for Pigs 1–5. Control specimens (NSC, IPC, and IDC) were procured from Pig 2. *a*-SMA, *a*-smooth muscle actin; CK, cytokeratin; DAPI, 4′, 6-diamidino-2-phenyllindole; UP3A, uroplakin 3A.

**FIGURE 6:**
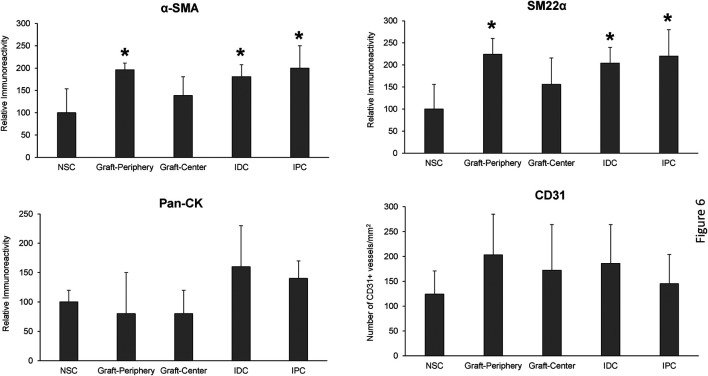
**Histomorphometric evaluations of tissue-specific proteins in neoconduits and control groups.** Quantitative analyses of marker expression in neotissue regions at 12 weeks post-op and control cohorts following immunohistochemical staining. Values are presented as means ± SD. NSC, nonsurgical controls; IPC, internal proximal controls; IDC, internal distal controls. Results from all groups were analyzed with Kruskal–Wallis and post hoc Dunn’s tests. *p < 0.05 relative to respective NSC levels. *N* = 5 samples per data point from all swine.

Regenerated and control tissues were next evaluated by IHC (Figure 5) and histomorphometric (Figure 6) analyses to assess the degree of *de novo* tissue maturation achieved at implant sites. Pan-CK + epithelia were observed throughout the entire length of regenerated tissues with the level of immunoreactivity similar between all experimental cohorts. In addition, UP3A positivity was also evident in the apical layers of all regenerated epithelia providing evidence for the formation of mature urothelium in neoconduits. Smooth muscle bundles expressing contractile proteins, *a*-SMA and SM22α, were detected in both central and peripheral areas of the *de novo* ureteral wall in all specimens. However, the expression pattern of these markers was discontinuous along the circumference of the neotissue interior suggesting muscle formation was underdeveloped at this timepoint. Relative immunoreactivity of *a*-SMA and SM22α was found to be significantly elevated in the graft-periphery, IPC and IDC segments in respect to the NSC group consistent with histological findings of smooth muscle hypertrophy and hyperplasia secondary to ureteral obstruction. De novo innervation and vascularization processes were also apparent in all neotissues as characterized by the presence of S100+ nerve fibers and blood vessels lined with CD31^+^ endothelial cells, respectively. Taken together, these results demonstrate that BLSF grafts support the formation of innervated, vascularized ureteral tissues with urothelial and smooth muscle components.

**FIGURE 7:**
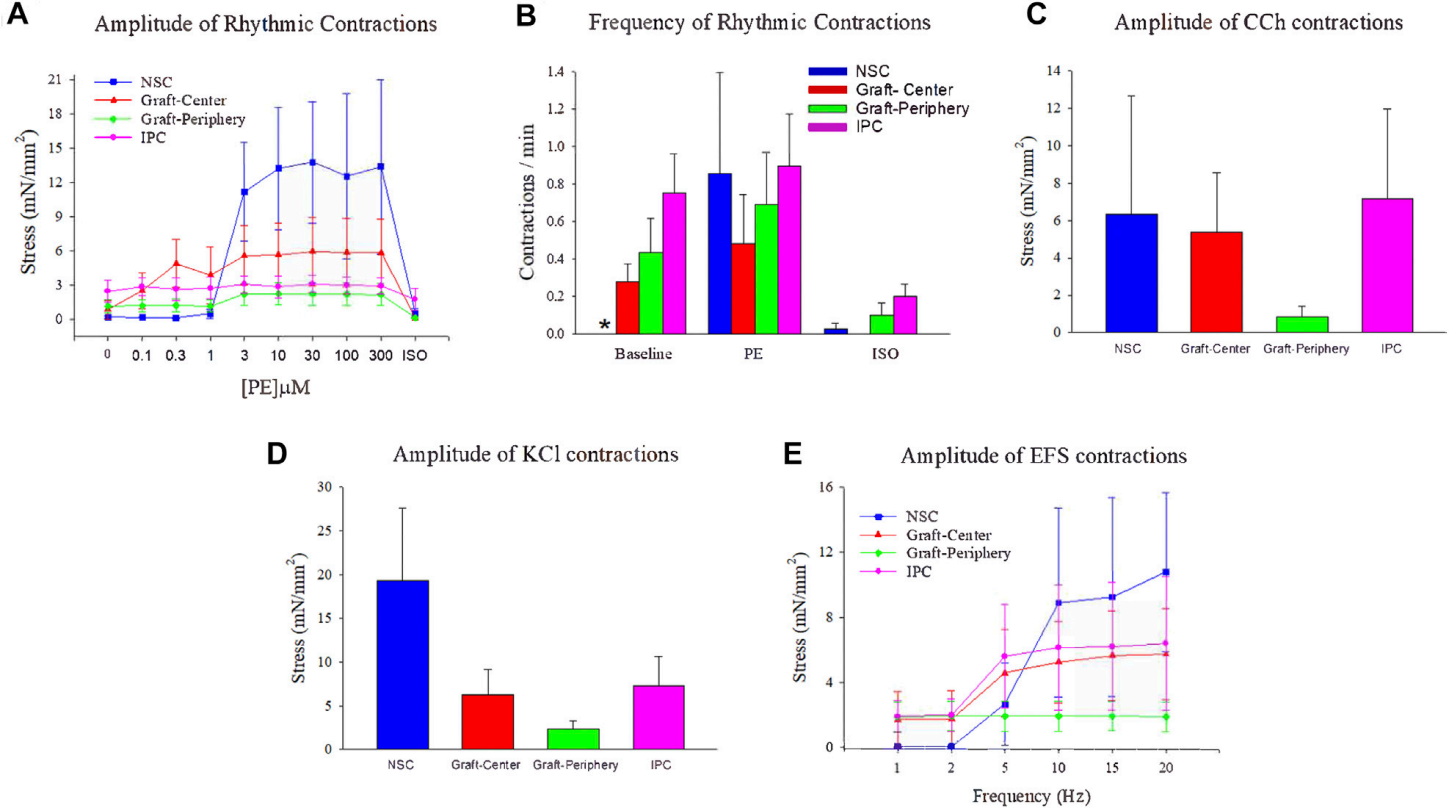
***Ex vivo* contractile and relaxation responses in experimental groups.** Quantitative assessments of contractile and relaxation responses in ureteral muscle tissue from NSC (blue bars and plots), graft-center (red bars and plots), graft-periphery (green bars and plots), and IPC segments at 12 weeks post-op. **(A)** The amplitude of rhythmic activity was measured before and after administration of increasing concentrations of phenylephrine (PE) followed by isoproterenol (ISO, 10 µM). **(B)** The frequency of spontaneous activity under baseline conditions, as well as the frequency of evoked activity after PE (300 µM) and (ISO, 10 µM), were compared among each segment. **(C–E)** The amplitude of ureteral evoked responses induced by **(C)** carbachol (CCh, 10 µM), **(D)** KCl (120 mM), and **(E)** electrical field stimulation (EFS). For all panels, values are displayed as means ± SE with *N* = 4 per data point including specimens from Pigs 1–4. Results from all segments were analyzed with Kruskal–Wallis and post hoc Dunn’s tests. *p < 0.05 relative to respective IPC levels in **(B)**. NSC, nonsurgical controls; IPC, internal proximal controls.

*Ex vivo* tissue bath analyses were performed to quantify the contractile/relaxation responses of control and regenerated ureteral tissues (Figure 7). Following tissue equilibration, spontaneous rhythmic contractions were observed in all experimental segments except NSC. The frequency of spontaneous activity was significantly higher in IPC specimens compared to NSC under baseline conditions, although the amplitude of spontaneous activity was not different among segments (Figures 7A,B). Following administration of phenylephrine, rhythmic contractions became prominent in ureteral tissues from all segments, while the exposure to ISO reduced these contractions in each segment (Figures 7A,B). Ureteral tissue responded to CCh (Figure 7C) and KCl (Figure 7D) stimulation with a sustained contraction in all segments. Nerve-mediated contractions of variable amplitudes were recorded in response to EFS in both control and reconstructed neotissues. Although the graft-periphery region generated less force compared to the other segments in response to CCh, KCl and EFS, there were no significant differences in contractile responses among segments (Figure 7E). These data provide evidence that ureteral reconstruction with tubular BLSF grafts leads to functional smooth muscle formation with contractile and relaxation properties.

## Discussion

Major ureteral reconstruction necessitates the use of tissue replacements to repair damaged ureteral segments and preserve upper urinary tract continuity. The goal of this study was to investigate the ability of BLSF grafts to promote functional repair of tubular ureteral defects in a porcine model. We chose to evaluate acellular scaffold configurations for ureteral tissue engineering since they offer particular advantages for clinical translation in comparison to cell-seeded counterparts including “off-the-shelf” utility and lack of secondary surgeries for procurement of patient-derived cells for construct seeding. Adult swine were used as a model species since the porcine ureter is of sufficient diameter to accommodate scaffold anastomosis, organ anatomy is similar to humans, and developmentally mature animals display minimal growth-related changes in ureteral dimensions during the experimental period (Greca et al., 2004; de Jonge et al., 2015).

Overall, our data provide evidence that tubular BLSF conduits in combination with transient stenting support intrinsic regenerative processes sufficient to produce innervated, vascularized ureteral tissues with contractile/relaxation functionality. However, a major limitation of the current scaffold design and surgical approach was the development of severe hydronephrosis, hydroureter and renal damage secondary to ureteral obstruction following stent removal. Ureteral occlusion was a consequence of stricture formation in central regions of neoconduits as well as extrusion and poor degradability of bulk scaffold remnants into the distal ureter. These results are supported by observations that ureteral dilation was present in both proximal and distal segments relative to the original implantation site in the majority of swine. Transient stenting of tubular tissue engineered constructs has been previously used to mitigate stenosis and hydronephrosis in reconstructed ureters presumably by reinforcing the mechanical integrity of the neotissue lumen during early stages of tissue remodeling (Zhang et al., 2012; Liao et al., 2013). However, histological and IHC evaluations confirmed that central neotissue areas supported by BLSF grafts were immature at sacrifice relative to peripheral neotissues and NSC suggesting longer periods of stenting may be necessary for complete regeneration and stricture prevention. Indeed, the duration of stent deployment has been shown to play a major role in determining the risk of stricture occurrence in reconstructed visceral hollow organs (Takimoto et al., 1994; Gundogdu et al., 2021). In addition, the *in vivo* degradation profile of BLSF matrices was also found to be insufficient for maintaining upper urinary tract function following stent removal.

Previous *in vivo* studies of porous silk foams have demonstrated that the initial silk fibroin content and pore size significantly influence the rate of scaffold degradation and degree of host tissue ingrowth (Wang et al., 2008). These data suggest that the degradation rate of the foam compartment of BLSF grafts may be increased by reducing the concentration of silk fibroin used during casting or by enhancing pore size via modulation of salt porogen diameter. In addition, *in vivo* degradation kinetics of silk fibroin films have been reported to be significantly dependent on the degree of *ß*-sheet crystallinity which can be temperature controlled during water vapor annealing processes (Ghezzi et al., 2016). Future experiments will focus on optimization of BLSF scaffold fabrication parameters to enhance degradation kinetics by implementing selective changes in the casting process for both the film and foam layers. Specifically, we predict that reductions in the initial silk fibroin content of the foam compartment in combination with increases in pore size via modulation of porogen diameter will result in more rapid *in vivo* degradation of the BLSF graft. We also anticipate that bulk BLSF matrix degradation can be further improved by reducing the level of *ß*-sheet crystallinity in the film layer by deploying water vapor annealing at 25°C for film construction as previously reported (Ghezzi et al., 2016).

Differential wound healing outcomes between peripheral and central neotissue areas suggest that *de novo* tissue formation occurs from ingrowth of host ureteral tissues adjacent to the anastomotic borders of the implantation site. Host tissues appear to primarily propagate along the exterior of the graft wall from both proximal and distal directions toward the interior resulting in luminal extrusion of the bulk matrix while promoting defect consolidation. By utilizing the film layer of BLSF grafts as a substrate, the invading neomucosa is potentially shielded from noxious urinary components in the foam compartment until epithelialization is complete. Indeed, BLSF grafts were capable of promoting multi-layered, urothelial formation in neotissues with prominent UP3A and pan-CK protein expression. This pattern of constructive remodeling is similar to our previous observations using BLSF scaffolds for bladder and esophageal tissue repair (Affas et al., 2019; Gundogdu et al., 2021).

Ureteral peristalsis plays a crucial role in transporting urine from the renal pelvis toward the bladder for storage and expulsion. Ureteral neoconduits supported by BLSF grafts contained circular smooth muscle layers with the ability to produce spontaneous and phenylephrine-induced, rhythmic contractions that could be attenuated by isoproterenol. These findings demonstrate myogenic activity in the neotissues that may underlie peristalsis *in situ*, and functional *a*- and *ß*-adrenoceptors in regenerated smooth muscle that can modulate urine propulsion. Regenerated tissues also showed a propensity to contract in response to KCl and CCh stimulation, indicating a fully intact contractile machinery capable of activation by membrane depolarization and muscarinic receptor signaling, respectively. Our findings also revealed that neoconduits were capable of contracting in response to EFS, thus indicating that excitatory neurotransmitters are released from functionally intact intrinsic nerves in implant regions.

## Conclusion

The findings from our current report reveal that acellular, tubular BLSF scaffolds are permissive for ureteral tissue formation when deployed in tandem with transient stenting. All neotissues were composed of vascularized, innervated epithelial and muscular components capable of contractile and relaxation responses with minimal inflammatory reactions. Stented neoconduits were able to maintain upper urinary tract function during initial stages of implantation. However, future improvements in our graft technology are necessary to mitigate stricture formation and enhance *in vivo* scaffold degradation following stent removal to prevent long-term ureteral obstruction. In summary, BLSF biomaterials represent emerging platforms for tubular ureteroplasty and may offer a functional replacement for conventional autologous grafts following further optimization.

## Data Availability

The raw data supporting the conclusions of this article will be made available by the authors, without undue reservation.
